# Oxalate decarboxylase uses electron hole hopping for catalysis

**DOI:** 10.1016/j.jbc.2021.100857

**Published:** 2021-06-05

**Authors:** Anthony J. Pastore, Ruijie D. Teo, Alvaro Montoya, Matthew J. Burg, Umar T. Twahir, Steven D. Bruner, David N. Beratan, Alexander Angerhofer

**Affiliations:** 1 Department of Chemistry, University of Florida, Gainesville, Florida, USA; 2 Department of Chemistry, Duke University, Durham, North Carolina, USA

**Keywords:** oxalate decarboxylase, X-ray crystallography, long-range electron transfer, tryptophan pair, LRET, long-range electron transfer, OxDC, oxalate decarboxylase, PDB, Protein Data Bank, VIE, vertical ionization energy

## Abstract

The hexameric low-pH stress response enzyme oxalate decarboxylase catalyzes the decarboxylation of the oxalate mono-anion in the soil bacterium *Bacillus subtilis*. A single protein subunit contains two Mn-binding cupin domains, and catalysis depends on Mn(III) at the N-terminal site. The present study suggests a mechanistic function for the C-terminal Mn as an electron hole donor for the N-terminal Mn. The resulting spatial separation of the radical intermediates directs the chemistry toward decarboxylation of the substrate. A π-stacked tryptophan pair (W96/W274) links two neighboring protein subunits together, thus reducing the Mn-to-Mn distance from 25.9 Å (intrasubunit) to 21.5 Å (intersubunit). Here, we used theoretical analysis of electron hole-hopping paths through redox-active sites in the enzyme combined with site-directed mutagenesis and X-ray crystallography to demonstrate that this tryptophan pair supports effective electron hole hopping between the C-terminal Mn of one subunit and the N-terminal Mn of the other subunit through two short hops of ∼8.5 Å. Replacement of W96, W274, or both with phenylalanine led to a large reduction in catalytic efficiency, whereas replacement with tyrosine led to recovery of most of this activity. W96F and W96Y mutants share the wildtype tertiary structure. Two additional hole-hopping networks were identified leading from the Mn ions to the protein surface, potentially protecting the enzyme from high Mn oxidation states during turnover. Our findings strongly suggest that multistep hole-hopping transport between the two Mn ions is required for enzymatic function, adding to the growing examples of proteins that employ aromatic residues as hopping stations.

Long-range electron transfer (LRET) is recognized as an essential feature of redox catalytic proteins (1, 2, 3, 4). Prominent examples include photosynthetic proteins (5, 6, 7) and the proteins that facilitate charge transfer in the respiratory chain (8), but there are many others (9). LRET between different redox cofactors in these proteins typically occurs by an electron tunneling mechanism, mediated by protein superexchange interactions. In some cases, redox active amino acids, particularly tyrosine and tryptophan, enable a multistep hopping mechanism (10, 11, 12, 13, 14). This mechanism involves the progress of the electron from donor to acceptor (or, equivalently, a hole from electron acceptor to electron donor), in a sequence of multiple electron transfer reactions. An electron hole is a quasi-particle and represents the lack of an electron from an orbital where it could exist.

In 2015, Gray and Winkler showed that approximately one-third of the protein structures in the Protein Data Bank (PDB) have putative redox chains of three or more residues linked to a surface-exposed tyrosine or tryptophan, and they suggested that hole hopping through these groups may serve as a protection mechanism from oxidative damage (10). More recently, Teo *et al.* (15) developed a kinetic model to describe multistep hopping transport through proteins. It allowed the theoretical identification of putative hole-hopping escape routes in cytochrome P450 monooxygenase (P450_BM3_ from *Bacillus megaterium*), cytochrome *c* peroxidase (Ccp1 from *Saccharomyces cerevisiae*), and benzylsuccinate synthase (BSS from *Thauera aromatica*) (15). It is interesting to note that the occurrence frequency of tryptophan and tyrosine in these proteins, *i.e.*, 1.1% to 2.4% for W and 2.6% to 4.8% for Y, is at or above the average across the tree of life (1.3% and 2.5% for W and Y, respectively) (16). Teo *et al.* (17) also used the model to describe electron or hole-hopping transfer between iron–sulfur clusters and DNA/RNA in the human primosome–DNA/RNA complex. This kinetic analysis can be used to analyze many kinds of biological electron transfer hopping networks. In this article, we describe a combined theoretical and experimental study of potential electron transfer pathways in oxalate decarboxylase (OxDC) of *Bacillus subtilis*. Our calculations point toward an efficient hole-hopping pathway between the N- and C-terminal Mn ions through a π-stacked tryptophan pair (W96/W274) located at the interface between two subunits in the hexameric quaternary structure of the protein. Experimentally, our site-directed mutagenesis experiments indicate that these residues play a prominent role in catalysis, enabling electron transport between redox cofactors on a catalytically relevant time scale.

OxDC is a Mn-dependent enzyme in the cupin superfamily and is found in fungi and soil bacteria (18, 19, 20, 21). It is a stress-response enzyme in certain soil bacteria and is expressed under acidic conditions and translocated to the periplasm and the cell wall (21, 22, 23, 24). Oxalic acid is the most common low-molecular-weight dicarboxylic acid found in the soil, usually complexed with calcium (25, 26). As a plant metabolite it is part of the dietary intake of many mammals and because of its inherent toxicity presents a significant health risk (27, 28). It also plays a role in the pathogenicity of fungal plant diseases (29). Understanding the expression, gene regulation, structural organization, and catalytic mechanism of OxDC and related oxalate-degrading enzymes is expected to advance efforts to enhance fungal resistance in crop plants and to lower their oxalate concentration (30, 31). Other potential applications are being explored in the areas of oxalate scale remediation (32, 33), novel therapeutics for hyperoxaluria and kidney stones (34, 35), and bioengineered probiotic gut bacteria (36, 37). Efforts have also been made to modify the protein to improve its catalytic activity at normal pH (38).

The best characterized isozyme of OxDC is from *B. subtilis*, which can be conveniently overexpressed in *Escherichia coli* (39). It catalyzes the heterolytic cleavage of the relatively inert carbon–carbon bond of the oxalate mono-anion, a unimolecular disproportionation reaction that is nominally redox neutral. Yet, the presence of dioxygen is obligatory for catalysis, and O_2_ is generally considered to act as a co-catalyst (39). The bicupin enzyme requires Mn ions coordinated in the center of each cupin fold by three histidines and one glutamate (40, 41, 42). Although both Mn-binding sites need to be occupied for full activity (43), it is generally accepted that only the N-terminal Mn site acts as the active site for catalysis (44). This conclusion is based on site-directed mutagenesis experiments involving the flexible SENST161-165 loop that gates substrate access to the N-terminal site (45, 46), and at a second-shell tryptophan residue, W132 (47, 48), in combination with X-ray and kinetic isotope effect data (44, 49, 50). The mechanistic role of the Mn ion bound to the C-terminal domain is unknown. However, it is necessary for catalysis (43), and it is more difficult to oxidize than the N-terminal Mn ion (51).

Figure 1*A* shows the literature mechanism of OxDC in black with the proposed extension based on the work described in this contribution in gray. Figure 1*B* illustrates the potential electron transfer (hole-hopping) pathway between the N- and C-terminal Mn ions across the W96/W274 tryptophan pair. Enzymatic activity of OxDC is strongly pH dependent, with a maximum at around pH 4.0 (49, 52). The substrate is generally considered to be the mono-anion of oxalate, C_2_HO_2_^−^, which has a p*K*_a_ of 4.3 (52). Only about 16% of the Mn in enzyme preparations poised at low pH is in the +3 state, essentially all located at the N-terminal site (51). The pH dependence of the Mn(III) EPR signal closely follows the pH dependence of the catalytic activity, which suggests that Mn(III) is the driver of catalysis (51). It is generally accepted that dioxygen is needed for catalysis, and most mechanistic schemes in the literature presume it is bound directly to the N-terminal Mn as a superoxide, indicated by the letter X in Figure 1*A* (44). However, experimental evidence for the existence of a superoxide-bound Mn(III) in OxDC is still lacking. Moreover, the existence of such a complex under turnover conditions would interfere with the proposed intermediate oxalate radical, and one should expect it to lead to a two-electron oxidation of the substrate yielding two equivalents of carbon dioxide and one of hydrogen peroxide. Superoxide was indeed observed by EPR spin trapping during turnover, together with an intermediate carbon dioxide radical anion (53). However, the trapping ratio of these two radicals distinctly changes in the T165V mutant that favors the open conformation and strongly suggests that the two radicals originate from two different locations in the protein (53). We speculated, therefore, that oxygen might bind to the C-terminal Mn ion (see the gray part of the mechanism in Fig. 1*A*) (53). This would protect the oxalate radical at the N-terminal site from further oxidation and explain the rather low rate of oxidase activity of ∼0.2% of all turnovers (21, 39). However, this hypothesis requires a LRET pathway for the electron withdrawn from the substrate to make its way to a dioxygen bound at the C-terminal cupin domain. As we demonstrate here, such a hopping pathway does indeed exist *via* the π-stacked W96/W274 pair within the hexameric cluster found in the reported OxDC crystal structures (see Fig. 1*B*).

**Figure 1:**
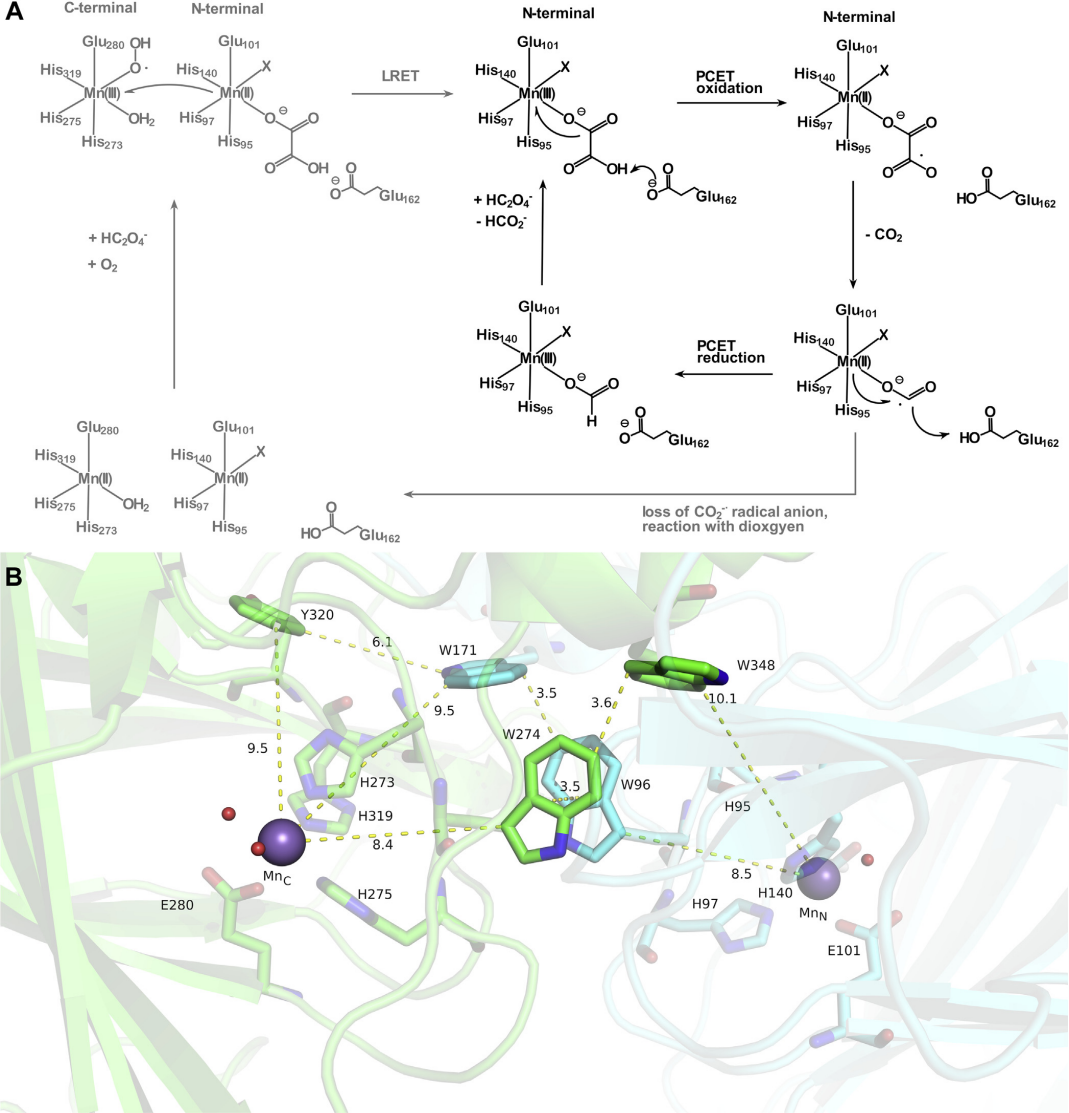
**Mechanistic proposal for OxDC catalysis.***A*, Schematic of the proposed catalytic mechanism of OxDC. The literature mechanism, *e.g.*, from ref. (24, 32), is shown in *black* and does not involve the C-terminal Mn ion. The extension proposed in this contribution is shown in *gray*. The letter X indicates the binding site assigned to dioxygen in the literature mechanism. *B*, W96/W274 π-stacked dimer in WT OxDC taken from the low pH structure (Protein Data Bank: 5VG3). Mn_C_ is the C-terminal Mn ion, Mn_N_ the N-terminal one. Other redox active residues potentially involved in hole transfer are the auxiliary tryptophans W171 and W348 and tyrosine Y320.

To test the hypothesis of W-mediated hopping transport in OxDC, site-directed mutants were prepared for W96 and W274. In order to protect the quaternary structure, we used the aromatic amino acid phenylalanine, which we hypothesized would maintain a π-stacking interaction with the neighboring indole, while disrupting the hole-hopping chain due to its higher reduction potential (54, 55). We find, indeed, that the W→F mutations significantly depress catalytic activity while the corresponding W→Y mutations partially rescue catalysis. Replacement of the phenylalanine with tyrosine was used as a control experiment. Since tyrosine has a redox potential similar to that of tryptophan it was expected to rescue activity (56). Hopping pathway calculations (using EHPath) were carried out on the mutants to analyze expected electron transfer rates in the proteins. Since W and Y occur at a slightly elevated frequency in OxDC of 1.6% and 3.7%, respectively, compared with the general average, we also explored other electron transfer paths in the protein. X-ray crystallography was carried out on single crystals of the W96F and W96Y forms to determine the structure of the W mutant proteins. The W274 mutant proteins and the double mutants did not produce X-ray quality single crystals.

## Results

Intra- and intermolecular long-range electron hopping pathways within the hexameric quaternary assembly of OxDC were assessed using the EHPath program described in Experimental procedures (15, 17). The program considers the residues TRP, TYR, MET, and CYS as potential electron/hole-hopping sites and uses estimates for their reorganization energies and reduction potentials taken from the literature (57, 58). Distances between possible donor–acceptor partners are determined from the X-ray structure and used to estimate the corresponding electronic couplings and reorganization energies. Individual hole-hopping rates were calculated for each donor–acceptor pair using Equation 1. EHPath searches for the pathways that support the most rapid electron or hole hopping between targeted sites. Each path is ranked using the carrier’s (electron or hole) estimated average residence time in the hopping pathway. As discussed in ref. (17), the mean residence time provides the time scale that a mobile electron or hole resides in a particular pathway and is inversely related to the overall rate of charge flow from the initial donor to the final acceptor.

We were particularly interested in the possible role of the W96/W274 TRP pair in mediating long-range electron hopping between neighboring subunits. We assumed that the C-terminal Mn serves as the hole donor and that the N-terminal Mn serves as the hole acceptor. This assumption follows the recognition that the +3 oxidation state was observed on the N-terminal Mn ion in the presence of dioxygen and a coordinating carboxylate anion but not on the C-terminal Mn (51). The additional negative charge of the carboxylate (the substrate is the mono-anion of oxalic acid, C_2_HO_2_^−^) likely stabilizes the Mn(III) state, and one can estimate a potential of the Mn(II)/(III) couple at well below 800 mV *versus* the normal hydrogen electrode (59). On the other hand, the C-terminal Mn ion is isolated from the solution, and none of the published crystal structures of WT or mutant OxDC shows solute ligands other than water in addition to the usual three histidines and one glutamate (40, 41, 42, 46, 47, 50, 60, 61). Hence, that site will not show the same stabilization of Mn(III) that the N-terminal Mn experiences in the presence of substrate. We therefore estimated the potential of the C-terminal Mn(II)/(III) couple to be ∼300 mV higher than that of the N-terminal site in our hopping pathway calculations. This difference is consistent with experimental reduction potentials of Mn complexed with small carboxylates in aqueous solution (59). Hole-hopping pathways were calculated with the C-terminal Mn as the hole donor and the N-terminal Mn as the hole acceptor (see Table 1).

**Table 1:** EHPath calculations for WT and mutant OxDC

Mutant	Fastest pathway	Residence time [ms]	Rate [s^−1^]
WT (inter)	Mn_C_ – dimer(W96/W274)–Mn_N_	8.10	123
WT (intra)	Mn_C_’–Y284–Y281–W102–Mn_N_	735	1.29·10^−4^
W96F	Mn_C_–W274–W348–Mn_N_	32.8	30.5
W96Y	Mn_C_–W274–Y96–Mn_N_	8.37	119
W274F	Mn_C_–Y320–W171–W96–Mn_N_	52.9	18.9
W274Y	Mn_C_–Y274–W96–Mn_N_	9.27	108
W96F/W274F	Mn_C_–W171–W348–Mn_N_	98.3	10.2
W96Y/W274Y	Mn_C_–Y274–Y96–Mn_N_	9.27	108

Inter refers to hole hopping between neighboring protein subunits, and intra refers to hole hopping through the interior of a single subunit. Mn_C_ refers to the C-terminal Mn ion, assumed to be the hole donor, and Mn_N_ refers to the N-terminal one, the presumed hole acceptor. Please note that these Mn ions are on neighboring subunits of the protein. Mn_C_’ is the C-terminal Mn on the same subunit as the N-terminal Mn.

The direct Mn_C_ (C-terminal Mn on second subunit)–W274–W96–Mn_N_ (N-terminal Mn on first subunit) pathway through the W96/W274 dimer is predicted to be the fastest (smallest residence time, see Table 1). A potential intrasubunit pathway, Mn_C_’–Y284–Y281–W102–Mn_N_, is significantly slower with a predicted residence time of 735 ms. Mn_C_’ refers to the C-terminal Mn in the same subunit as Mn_N_.

In the hopping pathway calculations, the π-stacked W96/W274 dimer was treated as a single “super molecule” assuming a potential lowered by 100 mV to a value of 900 mV as compared with a single TRP residue. Other TRP residues were assigned a potential of 1.00 V based on values reported by Mahmoudi *et al.* (58). The lower estimate of the TRP pair is in line with observations for π-stacked guanine potential shifts (62, 63). The lack of solvent access to the tryptophan dimer creates an electrostatic environment that makes it likely that their true reduction potential is even lower (64), possibly facilitating even faster hole transfer than estimated in our analysis.

We find the fastest hole-hopping rate along the path that involves only two hops: (1) from the C-terminal Mn to the W96/W274 dimer and (2) from the dimer to the N-terminal Mn. The molecules involved in this pathway, and the pathways calculated for the mutants, are shown in Figure 1*B*. Note that the Mn-to-edge distances between the two Mn ions and the tryptophan indole rings are approximately 8.4 Å, well within the range for effective sub-ms electron transfer found in proteins (65). The planes of the two tryptophans are almost parallel to each other and separated by 3.5 Å, while the distance between their C3 carbons is ∼4.9 Å, and almost directly lined up along the hole-hopping path. The Mn-to-Mn distance across the subunit boundary measures 21.5 Å and is thus shorter than the distance through a single subunit, 25.9 Å. Of interest, the single W→Y mutants (W96Y and W274Y) have predicted hopping rates approximately the same as in the WT simulations, confirming our premise that replacing tryptophan with tyrosine will have little effect on the overall electron hopping rates, assuming that a proton acceptor is available to establish a neutral tyrosyl radical as the hopping intermediate (66). However, when one of the Trp residues is replaced by Phe (W96F and W274F), the hopping time grows by a factor of 4 to 6. We also find that the vertical ionization energy (VIE) for the F96/W274 dimer is 7.19 eV (VIE for the Y96/W274 and W96/W274 dimers are 7.09 eV and 6.94 eV, respectively), thus indicating a larger energy barrier for hole transfer from the C-terminal Mn to the F96/W274 dimer. These mutants use one of the auxiliary tryptophans, W171 or W348, as a detour and W274F also involves Y320 in its calculated fastest hopping pathway. In comparison, the W→F double mutant, W96F/W274F, is predicted to further slow the hopping rate, as the hole has to move through both auxiliary TRP residues. On the other hand, for the W→Y double mutant (W96Y/W274Y), a similar rate as in WT is predicted, even though the tyrosine pair was not represented as a supermolecule in our calculations.

In order to test these theoretical predictions, and to evaluate experimentally whether the intersubunit electron/hole transfer path is relevant for catalysis, both W96 and W274 were replaced by Phe and Tyr individually, and also as a pair. Michaelis–Menten kinetics were observed for all mutants except the W96F/W274F double mutant, which did not display any observable activity. Results of the activity assays for WT OxDC and the various tryptophan mutants are given in Table 2. In addition to *k*_cat_ and *K*_M_, we report the Mn content per monomer as determined by inductively coupled plasma mass spectrometry. Given the almost linear dependence of activity on Mn content (43), we report the catalytic efficiency, *ε* = *k*_cat_/*K*_M_, normalized by the Mn content of the subunits. This number allows for a more accurate comparison of the catalytic competence of the mutants. The last column in Table 2 shows this number normalized to the number found for WT. Indeed, both W→F single mutants are significantly impaired, showing only approximately 10% to 20% of the WT activity in W96F and W274F, respectively. Both mutants contain only about 0.6 Mn ions per subunit each and normalization by the number of Mn ions per subunit yields a more accurate picture of their catalytic competence.

**Table 2:** Michaelis–Menten kinetic parameters of WT and mutant OxDC

Mutant	*K*_M_ [mM]	*k*_cat_ [s^−1^]	Mn per unit	*k*_cat_/Mn [s^−1^]	*ε* [mM^−1^·s^−1^]	*ε*/*ε*_WT_
WT	33.3 ± 0.4	89.2 ± 1.4	1.93	46.2 ± 0.7	1.39 ± 0.03	1
W96F	16.0 ± 1.5	1.00 ± 0.03	0.55	1.82 ± 0.05	0.11 ± 0.01	0.082 ± 0.008
W96Y	3.7 ± 0.9	5.3 ± 0.9	1.34	4.0 ± 0.7	1.1 ± 0.3	0.8 ± 0.2
W274F	6.7 ± 0.3	1.10 ± 0.03	0.58	1.90 ± 0.05	0.28 ± 0.01	0.204 ± 0.009
W274Y	10.3 ± 3.1	23.9 ± 2.8	1.89	12.6 ± 1.5	1.2 ± 0.4	0.9 ± 0.3
W96F/W274F	n/o	<7.5·10^−3^	0.82	<9.1·10^−3^	n/o	n/o
W96Y/W274Y	5.6 ± 0.5	0.20 ± 0.01	0.33	0.61 ± 0.03	0.11 ± 0.01	0.079 ± 0.007

*ε* = *k*_cat_/*K*_M_, normalized by the Mn content per subunit. Errors are reported as the standard deviation of the mean from triplicate measurements; n/o stands for not observed.

The experimental results indicate that both W→F single mutations reduce *k*_cat_ by almost two orders of magnitude. Some of this reduction can be associated with the relatively low Mn incorporation. However, after normalization of the catalytic efficiency by the Mn ions per subunit, their activity remains significantly lower than that of the WT enzyme. The double mutant W96F/W274F does not show any detectable activity in our hands. As expected, the W→Y single mutations rescue catalytic efficiency to almost WT levels. The W96Y/W274Y double mutation shows very low activity comparable with that of W96F, yet it is still improved from the phenylalanine double mutant level where no activity was detected. Table 1 also indicates that the hole transfer rate for the WT (123 s^−1^) is 14% larger than that for the W96Y/W274Y double mutant (108 s^−1^), whereas the catalytic efficiencies provide a difference of around an order of magnitude (Table 2). As the crystal structure of the double mutant was not solved in this study and could not be used in our EHPath calculations, we hypothesize that the double W→Y mutation weakens the π–π stacking of the aromatic side chains compared with the coupling in the reference π-stacked tryptophan pair, due to weaker orbital overlap between phenolic side chains of tyrosine compared with tryptophan. This change would lower the interresidue hole transfer rate, contributing to an overestimation of the calculated hole transfer rate for the W→Y double mutant in our modeled structure. Hence, the measurements confirm the observed trend of higher charge transfer rates when tyrosine replaces phenylalanine.

**Figure 2:**
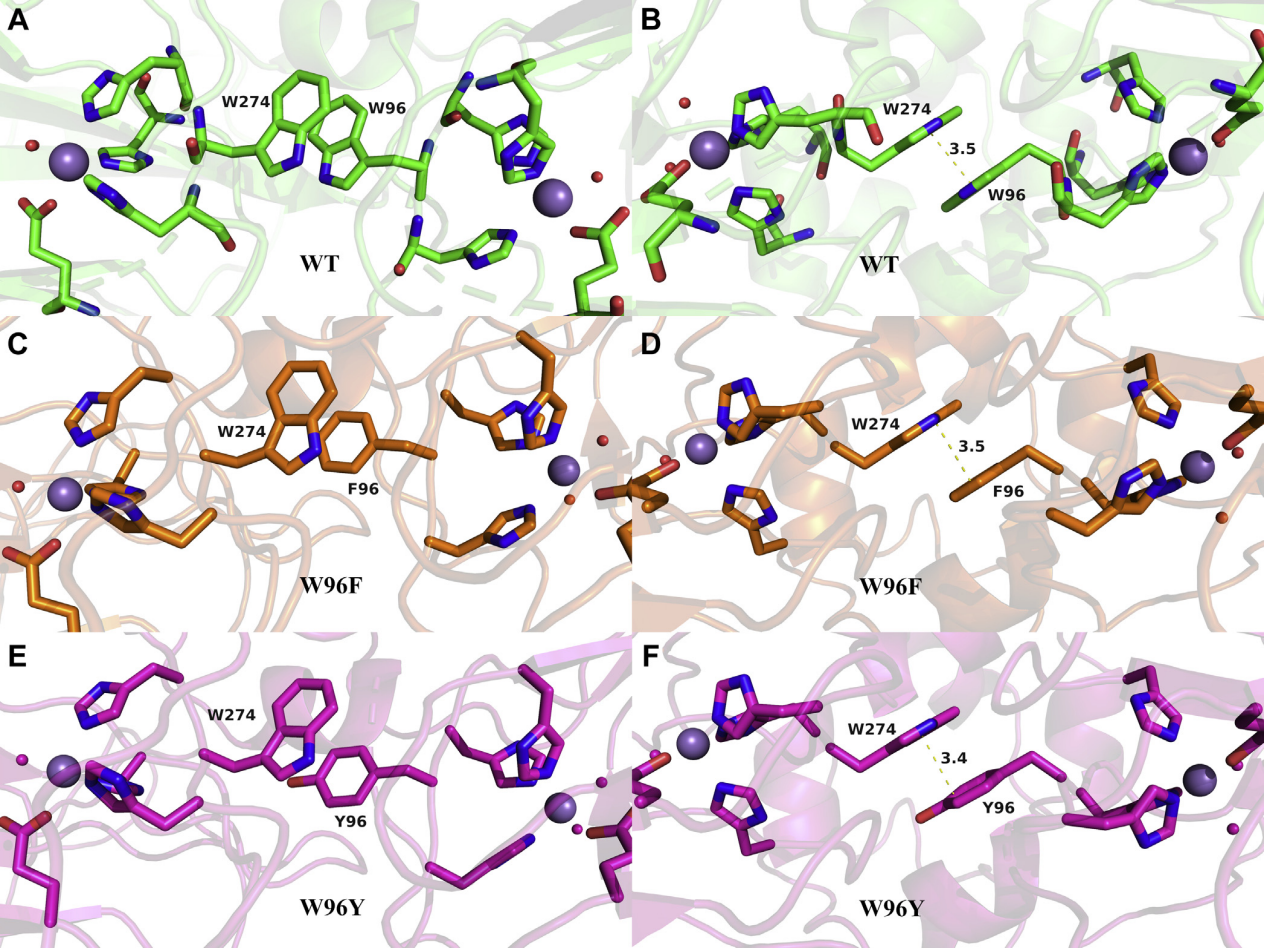
**Visualization of the π-stacked tryptophan dimer.** (*A* and *B*) W-W pair in WT OxDC. (*C* and *D*) W-F pair in W96F. (*E* and *F*) W-Y pair in the W97Y mutant enzyme. The two different viewpoints illustrate the ring systems (*left*) and the almost parallel aromatic planes in π-stacking mode (*right*). The C-terminal Mn (*large sphere*) is on the *left* and the N-terminal one on the *right*, both shown with their three HIS and one GLU ligands. W274 is therefore to the *left* and W(F,Y)96 on the *right side* in each panel.

Since the W96/W274 tryptophan pair is located at the interface between two neighboring subunits, we were concerned that these mutations might destabilize the quaternary structure of the enzyme presumed to be hexameric in solution (49). Comparison of the native PAGE gels of the mutant proteins after purification with that of WT suggests that all assemble in a similar manner (see Fig. S3). We attempted to grow single crystals of the W96 and W274 mutant proteins to examine the structural integrity of the mutant proteins further. This was successful for the two W96 variants (see Experimental procedures). These crystals diffracted to a resolution of 1.72 Å. Their crystal structures were solved and deposited in the RCSB PDB with the IDs 6TZP and 6UFI for W96F and W96Y, respectively. Comparison of the two structures with the low pH crystal structure, 5VG3, shows excellent agreement in the quaternary assembly as well as most other details. Significantly, the π-stacking of W274 with the substituted phenylalanine (W96F) and tyrosine (W96Y) aromatic ring system was conserved (Fig. 2). The average distance of the aromatic planes within each dimer differed only marginally by ∼ 0.1 Å or less. We therefore conclude that the loss of activity for the phenylalanine mutants is not due to a disruption of the quaternary structure.

Redox-active proteins are proposed to use chains of tryptophan and tyrosine residues to channel holes away from their active sites to protect the proteins’ integrity (15, 67, 68, 69, 70). It is well known that OxDC develops a reversible tyrosyl radical under turnover conditions, which is not linked to catalysis (71). Does the W96/W274 Trp pair participate in a protective function in OxDC in shuttling holes away from the N-terminal Mn? The C-terminal Mn appears to be the primary end point of the “bridge” between the two Mn ions. Yet, its reduction potential is higher than that of the N-terminal Mn, which makes it unlikely to serve as an outlet for a high oxidation state on the N-terminal site (51).

**Figure 3:**
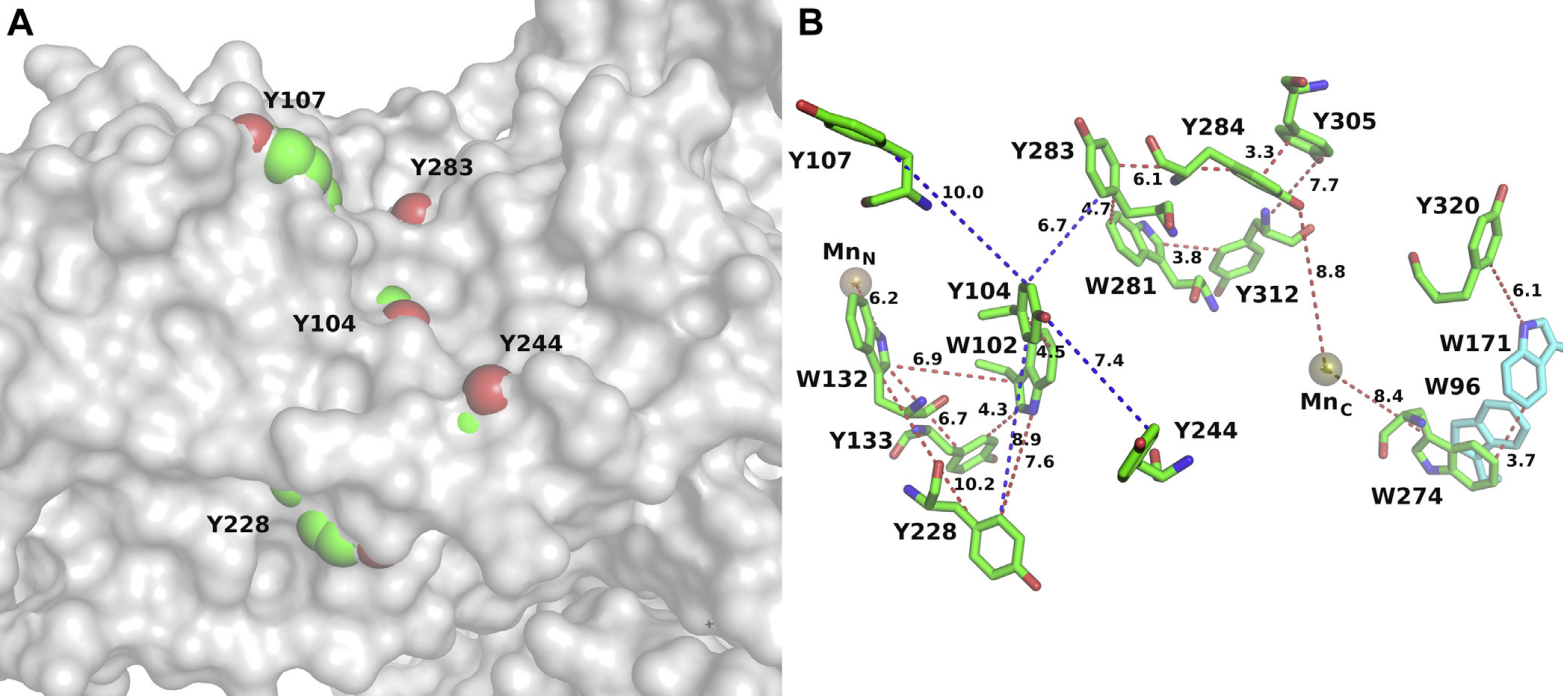
**Electron transfer pathways between aromatic amino acids in OxDC.***A*, Surface of chain A in *gray*. The five partially surface-exposed residues Y104, Y107, Y228, Y244, and Y283 stick out of the protein surface with their van der Waals spheres in element colors: C (*green*), O (*red*). *B*, Network of aromatic residues and nearest edge-to-edge distance for their aromatic rings. The N-terminal Mn is on the left and the C-terminal one on the *right*. The distances between the surface-exposed residues are shown with *blue dashes*. Distances between buried residues and between buried and surface-exposed residues are shown with *red dashes*.

However, is it possible that the W96/W274 pair is just the beginning of a longer multistep hole-hopping pathway that shuttles oxidative power away from the active site? With seven tryptophans and 14 tyrosines of 385 residues per subunit, OxDC contains a large number of potentially redox-active amino acids, similar to other proteins that were shown to have protective electron transfer chains (15). To investigate this question further, we carried out additional hopping pathway calculations to discover electron transfer from either Mn-binding site to aromatic residues at the surface of the protein, specifically Y107, Y228, and Y244, which are partially exposed, as well as to Y283, which has an oxygen atom exposed at the surface (Fig. 3). Y320 is surface exposed only for individual subunits. However, it is covered up by a loop from the neighboring subunit where the subunits connect at the corners of the triangles of the hexamer. Figure 3*A* shows the surface of subunit A in gray with the van der Waals surfaces of the five partially exposed residues (colored by element) protruding through the protein surface. Figure 3*B* shows the networks of nearest edge-to-edge distances between the aromatic residues that make up the predicted hopping transport network. Distances between surface-exposed tyrosines are shown with blue dashes, whereas distances of aromatic residues within the protein are shown with red dashes. The corresponding numbers are given in Å units.

The fastest hopping pathways that lead from the N- and the C-terminal Mn to surface-exposed residues are tabulated in Table 3. For the N-terminal Mn species, the key step is hole hopping to W132. W102 may also serve as a hopping site, and it connects W132 with both Y104 and Y228. There are several hopping paths that lead from the N-terminal Mn to the protein surface, ending either at Y104 or Y107, which are both partially surface exposed and within electron hopping distance of each other, or at Y228 (Fig. 3*B*). All of these pathways have very similar predicted hopping rates. Y104 is surface exposed but within a hopping distance of two other surface-exposed residues, Y107 and Y228. The predicted hopping rates are almost 20 times slower than the calculated rates from the N- to the C-terminal Mn species through the W96/W274 dimer, which is expected if the latter is important for catalysis. For the C-terminal Mn ion, we found several viable hopping pathways to the surface, primarily arriving on the partially surface-exposed Y283. However, since Y283 is in electron hopping distance to Y104 the pathways from both Mn centers may merge there. The distributor for the C-terminal charge transfer to the protein surface appears to be Y284. We also included Y320 as a potential surface-exposed residue in our calculations since it is on the surface of an individual protein subunit. However, in the hexameric form, this residue is covered by a neighbor subunit due to its linkage at the corners of the triangles. For the C-terminal Mn distribution chain, the hopping distances are generally shorter compared with the network for the N-terminal Mn. Hence, their predicted residence times are shorter. Yet, they are still a factor of 7 longer (slower) than the predicted hole transfer from the C-terminal to the N-terminal Mn. This means that, when the C-terminal Mn assumes a high oxidation state, Mn(III), it will readily oxidize the N-terminal Mn rather than become reduced by electron transfer from the surface. Significantly, when Mn(III) is formed on the N-terminal Mn ion, the kinetic pathway for its reduction from surface residues is even slower. In essence, the N-terminal Mn ion functions as a hole sink. These theoretical observations support our hypothesis of the catalytic competency of hole hopping through the W96/274 dimer.

**Table 3:** Predicted fastest hopping pathway leading from the Mn ions to the surface

Mn location	Fastest pathway	Residence time [ms]	Rate [s^−1^]
N-terminal	Mn_N_–W132–Y133–W102–Y104	150	6.67
N-terminal	Mn_N_–W132–W102–Y104	151	6.62
N-terminal	Mn_N_–W132–Y228	153	6.54
N-terminal	Mn_N_–W132–W102–Y104–Y107	154	6.49
N-terminal	Mn_N_–W132–W102–Y228	155	6.45
N-terminal	Mn_N_–W132–W102–Y104–Y228	165	6.06
C-terminal	Mn_C_–Y284–Y283	43.4	23.0
C-terminal	Mn_C_–Y284–Y283–Y104–Y244	59.9	16.7
C-terminal	Mn_C_–Y284–Y305–Y312–W281–Y283	65.6	15.2
C-terminal	Mn_C_–W274–W171–Y320	70.5	14.2

EHPath calculations were performed on WT OxDC.

## Discussion

The π-stacking of the W96/W274 tryptophan pair that stabilizes the quaternary structure of OxDC was noticed early on by Just *et al.* (41) in their discussion of the structure of the closed conformation, PDB ID 1UW8. The proximity of this pair with the Mn-coordinating histidines, H95, H97, H273, and H275, was also noted. The authors proposed that this structural motif was responsible for transmitting structural changes from one Mn-binding site to the other, but they did not consider the possibility of charge transfer through the tryptophan pair (41). Speculation about a possible LRET pathway between the N- and C-terminal Mn started with the recognition that the two key intermediates, the carbon dioxide radical anion and the superoxide radical anion, originate at different locations in the protein (53). The W96/W274 tryptophan pair was immediately seen as a potential candidate for a charge transfer waypoint within the quaternary structure prompting us to conduct the current study in a two-pronged approach, *i.e.*, a theoretical analysis of the potential electron/hole transfer pathways in the protein coupled with site-directed mutagenesis of the two tryptophans in question. Our EHPath calculations demonstrated an efficient hole-hopping channel between the two Mn ions in OxDC through the W96/274 pair. Site-directed replacement of tryptophan by phenylalanine was used to inactivate LRET while replacement with tyrosine was used as a potential positive control owing to its similar reduction potential compared with tryptophan. X-ray crystallography was used to confirm that the quaternary structure was not significantly disturbed by the mutations.

One of our concerns was the possibility that a mutation of a residue in the second coordination shell of the N-terminal Mn ion could perturb its electronic structure and thereby introduce additional effects on the thermodynamic or kinetic behavior of the active site. In fact, Zhu *et al.* (48) used kinetic isotope effect measurements to show that a hydrogen bond between the second shell tryptophan (W132) and the first-shell ligand E101 of the N-terminal Mn ion affects the transition state structure and enzyme activity. This behavior may arise from changes in the midpoint potential of the N-terminal Mn(II)/Mn(III) couple in the W132F mutant enzyme. Although W96 can be argued to be a second shell residue neighboring the first shell residues H95 and H97, it points away from the active site, unlike W132. W96 is therefore not within hydrogen-bonding distance of any of the first shell ligands. Moreover, the distance between the N-terminal Mn ion and the center of mass of W132 is 7.7 Å, whereas the corresponding measurements for W96 and W274 yield distances of 10.0 and 12.1 Å, respectively (see Fig. S1). A direct effect of the W96F or W274F mutation on the energetics of the transition state is therefore unlikely. An electronic effect of the W274 replacements is even less likely, since that residue is in the second shell of the C-terminal Mn and far away from the N-terminal Mn active site. Yet, we find very similar effects on catalysis for both W96F and W274F mutations.

Our key finding is that a W→F mutation for either or both tryptophans of the π-stacked pair, W96/274, strongly suppresses enzymatic activity, whereas a W→Y mutation preserves activity. This is in accordance with the theoretical prediction that the dimer acts as a facile hole-hopping pathway between the two Mn ions in the quaternary structure of OxDC. Based on these findings, we put forward the hypothesis that the W96/274 TRP pair functions as a catalytically competent hole-hopping bridge between the two Mn centers. Tryptophan electron transfer bridges are found in many other proteins, including DNA photolyase (54), the methylamine utilization protein MauG (72), cytochrome P450 (73), and others (74). Electron transfer through Trp and Tyr in proteins usually takes place by hole hopping (10). For tryptophan, the most common intermediate state is that of the protonated cation, TRPH^+•^ (75), although the neutral radical, TRP^•^, has also been observed (76). Inspection of the W96/274 pair in 5VG3 shows H-bonding distances between both indole nitrogens and backbone oxygens of the neighboring subunits (see Fig. S2). W96 is H-bonded to A316, and W274 to GLY137. This provides additional stabilization of the quaternary structure beyond the π-stacking interaction. Given the hydrophobic environment for these residues, it is unlikely that any protons present can escape to the solvent, nor is it likely that water molecules enter the pocket on the relevant ms timescales. Any through-pair electron transfer is expected to utilize a tryptophan cation radical state, *i.e.*, a hole.

How reasonable is it that hopping transport through the W96/274 pair could be catalytically relevant? Mechanistic proposals in the literature (44) are based on the idea that the C-terminal site is not a catalytic center and mainly serves a structural role (41, 42), even though high-field EPR spectra revealed a strong pH dependence of the C-terminal Mn ion suggesting a more involved role in catalysis (77). Our data indicate that this view has to be revisited, but how can the C-terminal Mn participate in catalysis in a way that utilizes the tryptophan pair as a hole shuttle? Redox cycling experiments revealed the asymmetry of the reduction potentials of the two Mn centers identifying the N-terminal Mn as the preferred hole sink (51). Together with the absence of a flexible lid gating substrate access to the C-terminal site (41, 42) this is a strong argument against a sort of ping-pong mechanism where the hole would be carried back and forth between two active centers supporting catalysis. Rather, the directionality of hole transport suggests that the C-terminal Mn is the source of the hole needed at the N-terminal site for catalysis. Our working hypothesis, shown in Figure 1*A*, is that dioxygen binds to the C-terminal Mn ion providing the extra driving force for hole transfer from the C- to the N-terminal site. This proposal spatially separates the two radical intermediates, CO_2_^−^^•^ and O_2_^−^^•^, preventing them from reacting with each other, which would lead to a second oxidation process and overall oxidase activity. Our hypothesis is further supported by the observation that both radicals originate from different locations on the protein (53).

It is well known that OxDC acts as an oxidase during approximately 0.2% of all turnovers (39). These enzymatic misfires can be interpreted as either due to trapped dioxygen in the active site or the loss of the intermediate carbon dioxide radical anion into the solution. Free CO_2_^−^^•^ radical in solution is expected to react with dioxygen to generate carbon dioxide and hydrogen peroxide (46, 53). Once OxDC undergoes a rare oxidase event with substrate, its N-terminal Mn becomes reduced to the +2 state and needs to be recharged by an oxidant, presumably dioxygen or superoxide. The generation of Mn(III) at the N-terminal site follows binding of a small carboxylate anion, which may be the substrate itself (51, 78). The additional negative charge of the coordinated carboxylate provides the needed stability for Mn(III). For OxDC this presents a problem. If dioxygen binds first at the active site it has to wait for the substrate to bind before it can oxidize the Mn to initiate catalysis. This would trap the resulting superoxide in place committing the enzyme to oxidase activity. If the substrate binds first it blocks access for dioxygen to the active site. Our hypothesis resolves this problem by utilizing the C-terminal Mn ion for dioxygen binding with subsequent hole transfer to the N-terminal Mn ion. Even though the C-terminal Mn does not have a flexible loop to gate solvent access there exists a narrow channel with a “static” diameter of only 0.7 Å, too narrow for substrate but wide enough for small diatomic species such as dioxygen or superoxide (61). A subtle feature of our proposal is that LRET is not required for each turnover event but is only needed to recharge Mn(II) to Mn(III), *i.e.*, when the active site Mn gets reduced after an oxidase event.

Our proposal makes dioxygen a promoter of catalysis rather than a cocatalyst. At the same time, it explains why the enzyme does not act primarily as an oxidase by spatially separating the radical intermediates from each other. The observed drop of approximately one order of magnitude in enzyme activity upon modification of the electron transfer bridge between the two Mn centers provides the first robust evidence for such a redox role for the C-terminal Mn ion. However, there remain many open questions. In particular, the geometry and electronic structure of a C-terminal Mn complex with dioxygen that would necessarily precede LRET remains to be established.

The existence of charge transfer pathways from the Mn ions to the protein surface (see Table 3) points to the possibility that the enzyme has a safety valve when Mn(III) is generated in the presence of small carboxylates but in the absence of substrate. Moreover, this finding explains why it is possible to readily oxidize both Mn ions chemically with hexachloroiridate in solution (51). These considerations may provide insight into the observation of a radical side product in the reaction, which was identified as a tyrosyl radical but could not be associated with a specific TYR residue (71).

In conclusion, our experiments and theoretical analysis indicate that W96 and W274 are important for catalysis in OxDC. Replacing these tryptophan residues with phenylalanine leads to an approximate order of magnitude drop in catalytic efficiency, and this change is reflected in the hopping pathway analysis. When phenylalanine is replaced by tyrosine, activity is significantly restored. This experimental fact, coupled with the theoretical prediction of efficient hole hopping between the two Mn ions, lends strong support to the hypothesis that electron hopping between the C- and N-terminal Mn ions plays a central role in the catalytic mechanism of this enzyme. Moreover, we have identified a network of electron hopping pathways, emanating from the Mn ions, that may be used by the protein to protect itself against potentially damaging high-oxidation-state species arising during enzymatic turnover.

## Experimental procedures

### EHPath calculations

The rate constant *k* for each electron/hole tunneling step was calculated using a Marcus-like high-temperature nonadiabatic rate expression (3),

(1) $$K=\frac{2\text{π}}{\hbar }\;\left\langle V^{2}\right\rangle \frac{1}{\sqrt{4\text{π}{\mathrm{\lambda k}}_{\text{B}}T}}\text{exp}\left[\frac{-{\left(\text{Δ}G^{\text{°}}+\lambda \right)}^{2}}{4{\mathrm{\lambda k}}_{\text{B}}T}\right]$$

where *V* is the electronic coupling between donor and acceptor states, λ is the reorganization energy, $\Delta G^{{}^{\circ}}$ is the standard free energy change between the initial and final states, and *T* is the temperature (298 K).

The EHPath program finds the fastest multistep hopping pathways (15). The required electron transfer parameters *V*, *λ*, and $\Delta G^{{}^{\circ}}$ were obtained using a square-tunneling barrier model, Marcus’s two-sphere model (3, 79, 80), and the difference in the donor and acceptor redox potentials. The inner-sphere reorganization energy for the Mn(II)/(III) self-exchange reaction was taken from Johnson and Nelson (81). The effective radii of the Mn species and the electronic couplings between Mn ions were obtained from Rosso *et al.* (82).

### DFT calculations

We performed density functional theory calculations to calculate the VIE for the three dimers W96/W274, Y96/W274, and F96/W274. For each dimer, the atomic coordinates of the side chains were retained from the respective crystal structures. The α-carbons were replaced by methyl groups, and hydrogen atoms were added using Avogadro version 1.20 (83). Geometry optimization of the hydrogen atoms was performed using the TZVP basis set (84) and the range-separated exchange-correlation functional CAM-B3LYP (85). The resulting coordinates were used to calculate the VIE with the cc-pVTZ basis set and CAM-B3LYP functional. The ORCA package (86, 87, 88), version 4.2.1, was used for all density functional theory calculations.

### Protein expression and purification

Expression and purification of recombinant His_6_-tagged WT and mutant OxDC were carried out following published procedures (42, 45, 46, 47). Cells were grown to an optical density of 0.5 at 600 nm in Luria-Bertani broth at 37 °C followed by heat shocking at 42 °C for 15 min. After heat shocking, MnCl_2_ was added to the cells in Luria-Bertani broth until the concentration of MnCl_2_ reached 4.6 mM. Isopropyl β-D-1-thiogalactopyranoside (IPTG) was also added for a final solution concentration of 0.8 mM IPTG. Cells were grown for four more hours before being centrifuged at 6000 revolutions per minute for 18 min at 4 °C. Cell pellets were stored at −80 °C until further use.

Approximately 8 g (wet mass) of cell pellets were resuspended in 40 ml of lysis buffer (50 mM Tris-HCl, 500 mM NaCl, 10 mM imidazole at pH 7.5) and lysed by sonification. Cell lysate was incubated with nickel-NTA resin (ThermoFisher HisPur) for 2 h at 4 °C and washed with eight column volumes of wash buffer (20 mM Tris-HCl, 500 mM NaCl, 20 mM imidazole at pH 8.5). OxDC was collected from fractions as the resin was washed with elution buffer (20 mM Tris-HCl, 500 mM NaCl, 250 mM imidazole, at pH 8.5). Imidazole is removed through standard dialysis with 50 mM Tris-HCl and 500 mM NaCl at pH 8.5. Chelex resin from Bio-Rad was used to remove free metal cations in solution, and Amicon Centriprep YM-30 centrifugal filter units (EMD Millipore) with a 30-kDa molecular weight cut-off were used to concentrate OxDC. Protein concentration was determined by the Bradford assay (Pierce) (89). The protein was further purified by FPLC. The first step involved anion exchange with a HiTrap Q HP column (5 ml). Gel filtration with a Superdex 200 10/300 GL column (GE Healthcare Life Sciences) was performed immediately afterward taking and concentrating the protein from the fractions of the anionic exchange eluents. The W96F mutant was concentrated to 7.0 mg/ml before using the sitting drop method to set up crystal screening. The W96Y mutant was concentrated to 3.7 mg/ml before optimization of crystal screening, which was accomplished with the hanging drop method. Details for crystallization can be found in Table 4. Inductively coupled plasma mass spectrometry determination of metal content was performed at the University of Georgia Center for Applied Isotope Studies Chemical Analysis Laboratory.

**Table 4:** Crystallization conditions for W96 mutants

Mutant	Method	Concentration (mg/ml)	Protein condition (1 μl)	Reservoir drop condition (1 μl)	T (°C)	Time	Length (μm)
W96F	Sitting Drop	7.0	20 mM Tris-HCl, 80 mM NaCl, 10 mM arginine, 10 mM glutamate, pH 8.5	10% PEG 8000, 100 mM Tris-HCl, and 200 mM MgCl_2_ at pH 7.0	4	1 month	50
W96Y	Hanging Drop	3.7	20 mM Tris-HCl and 100 mM NaCl at pH 8.5	7% PEG 8000, 100 mM Tris-HCl, and 200 mM MgCl_2_ at pH 7.0	4	5 days	100

### Site-directed mutagenesis

Phenylalanine and tyrosine mutants of W96 and W274 were prepared on a PET32A vector with the YvrK gene for OxDC and built-in ampicillin resistance as described (50, 51). Primers were designed using the NEBaseChanger online interactive software. Primers were obtained from IDT DNA. Primers are listed in Table S1. Site-directed mutagenesis was performed with the Q5 Site-Directed Mutagenesis Kit from New England Biolabs. Mutagenesis was prepared in a nonoverlap extension method optimized from the kit. PCR products were treated with DpnI, kinase, and T4 ligase (from the kit) before transformation inside DH5α cells from New England Biolabs. DH5α cells from New England Biolabs were used to amplify plasmid DNA. The SV Plus Wizard miniprep kit used for isolating DNA was purchased from Promega. Sequencing was accomplished through Genewiz.

### Catalytic activity assays

Michaelis–Menten parameters for the decarboxylase activity of OxDC were determined by an end-point assay measuring the production of formate, as described (39, 41, 47). The first step involves incubation of enzyme in an Eppendorf 1.5-ml tube with 0.004% (m/v) Triton X-100, 612 μM ortho-phenylenediamine, 500 mM NaCl, 50 mM poly buffer (50 mM each of citrate, piperazine, Tris-HCl, Bis-Tris methane [2-bis(2-hydroxyethyl)amino-2-(hydroxymethyl)-1,3-propanediol]]) at pH 4.2 and varying concentrations of potassium oxalate at 25 °C for a total volume of 104 μl. The reaction is quenched by the addition of 10 μl of 1 M NaOH. Fifty-five microliters of this mixture is mixed with 945 μl of an aqueous solution that contains 0.0004% (m/v) formate dehydrogenase, 50 mM K_2_HPO_4_ (pH 7.8), and 1.5 mM NAD^+^ with incubation overnight at 37 °C. The amount of NADH produced is measured by its absorption at 340 nm, which can be correlated to the amount of formate produced. A standard plot is generated from known concentrations of formate to indirectly quantify how much formate was produced by linear regression. Kinetic parameters were determined by modeling the raw data with Michaelis–Menten kinetics using Qt Grace software.

### Crystallization and X-Ray structure solution

Crystallization conditions and statistical parameters for the solution of the X-ray structure can be found in Tables 4 and 5. X-ray diffraction data were collected at beam lines 21-ID-F and 21-ID-G for W96F and W96Y, respectively, with a wavelength of 0.9787 Å at the Life Sciences Collaborative Access Team (LS-CAT) facility, Argonne National Laboratory Advanced Photon Source (APS-ANL). Crystals were kept at 100 K during data collection. The initial data were collected with a MARMOSAIC 300 mm CCD detector. XDS was used for indexing (90). The unmerged data were treated with Aimless (CCP4) for scaling (91). Molecular replacement was accomplished using Phaser (92). The phasing model utilized was PDB ID 1UW8 (41). PHENIX (93) was used for refinement of data, whereas Coot (94, 95) was used for visualization of structures. Figures of the W96F and W96Y crystal structures were generated with PyMOL, version 2.4.2 (96). Details of the data collection and refinement parameters are given in Table 5.

**Table 5:** Statistics for W96 mutant structures

Data collection and refinement	W96F (PDB: 6TZP)	W96Y (PDB: 6UFI)
Space group	R 3 2	R 3 2
a, b, c (Å)	155.07, 155.07, 123.08	155.27, 155.27, 124.09
α, β, γ (°)	90, 90, 120	90, 90, 120
Resolution (Å)	30–1.71 (1.78–1.71)	31–1.72 (1.79–1.72)
Rsym or Rmerge	0.122 (1.27)	0.0889 (0.380)
I/σ(I)	19.3 (2.3)	30.6 (9.4)
Completeness (%)	99.9 (99.2)	99.8 (98.5)
Redundancy	14.3 (13.8)	22.2 (21.2)
Resolution (Å)	1.71	1.72
No. reflections	865,952 (81,901)	1,351,073 (126,689)
Unique reflections	60,461 (5948)	60,978 (5965)
Rwork, Rfree	0.161, 0.179	0.150, 0.166
R-merge, R-measure, R-pim	0.122 (1.26), 0.127 (1.31), 0.033 (0.349)	0.089 (0.380), 0.091 (0.390), 0.019 (0.084)
CC-1/2, CC∗	0.999 (0.814), 0.999 (0.947)	0.999 (0.978), 0.999 (0.995)
No. of atoms		
Protein	2734	3072
Ligand/ion	2	6
Water	252	244
B-factors		
Protein	16.01	14.26
Ligand/ion	12.39	25.85
Water	23.49	21.94
R.M.S. deviations		
Bond lengths (Å)	0.006	0.006
Bond angles (°)	0.84	0.92

Data in parenthesis is for the highest-resolution shell.

## Data availability

X-ray coordinates for the W96F and W96Y mutant OxDC proteins have been deposited with the RCSB PDB databank and are accessible under the IDs 6TZP and 6UFI, respectively. The EHPath.py code can be accessed and downloaded at https://github.com/etransfer/EHPath.

## Supporting information

This article contains supporting information (39, 42, 52, 97).

## Conflict of interest

The authors declare that they have no conflicts of interest with the contents of this article.
