# Supplementary material for: Oxalate decarboxylase uses electron hole hopping for catalysis
Source: J Biol Chem. 2021 Jun 5;297(1):100857. doi: 10.1016/j.jbc.2021.100857 (PMC8254039; doi:10.1016/j.jbc.2021.100857)
Supplement: Supplemental Figures S1–S3 and Table S1 [file mmc1.pdf]

Supporting Information for:
Oxalate Decarboxylase uses Electron Hole Hopping for Catalysis

Anthony J. Pastore, Ruijie D. Teo, Alvaro Montoya, Matthew, J. Burg, Umar T. Twahir, Steven D. Bruner, David N. Beratan, and Alexander Angerhofer

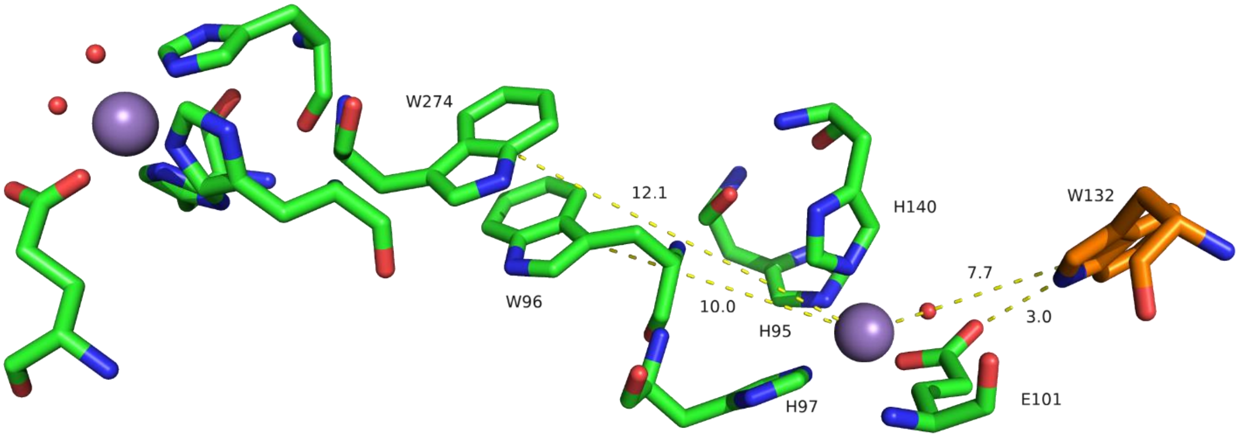

Figure S1: *Distances between the N-terminal Mn ion and the center of mass of W132, W96, and W274. The H-bonding distance between W132 and E101 is also shown. The distances are reported in Angstroms.*

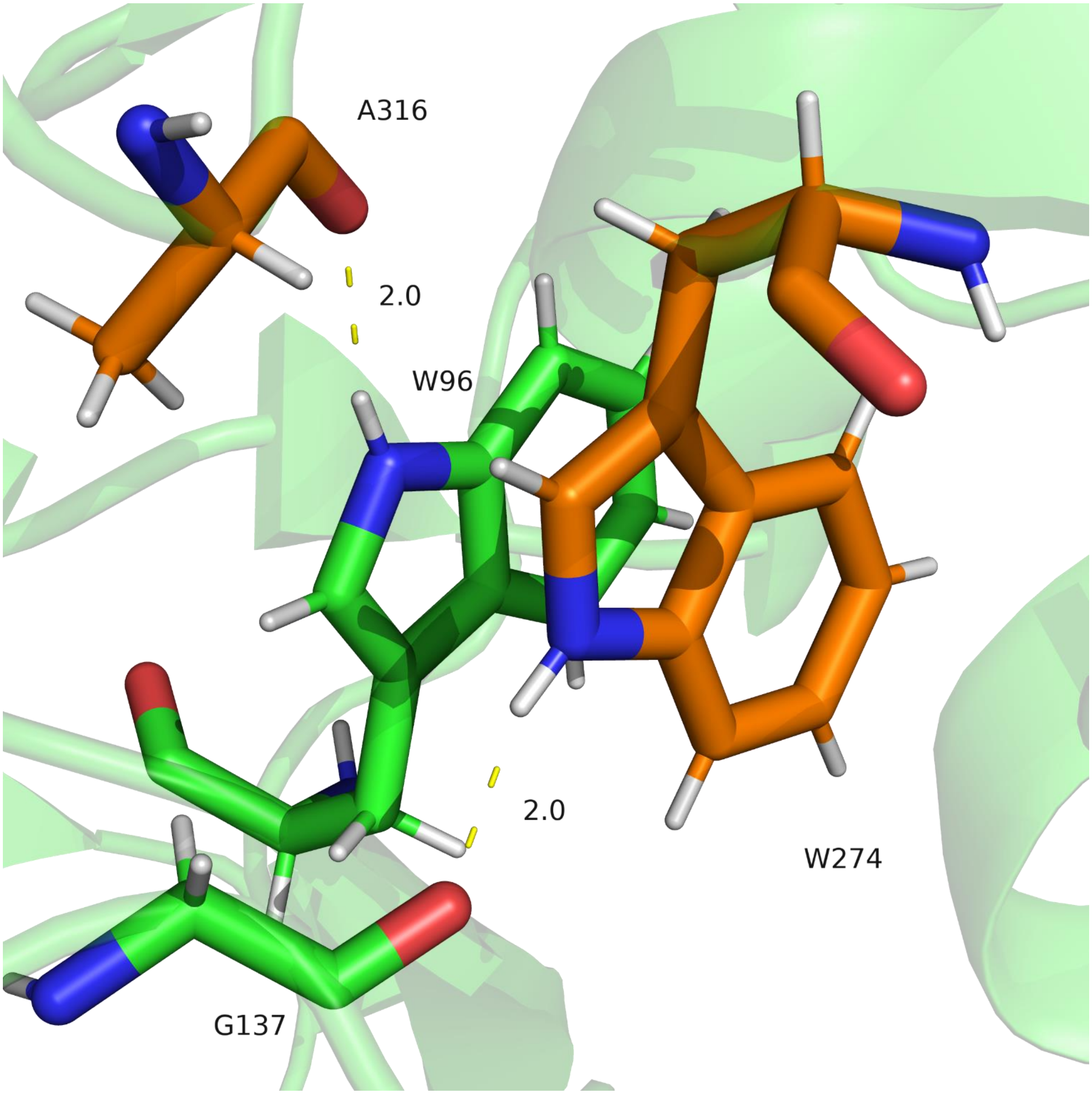

Figure S2: *H-Bonding between the TRP pair W96/274 and the backbone of the neighboring subunit. H atoms were added to 5VG3 using MolProbity (1).*

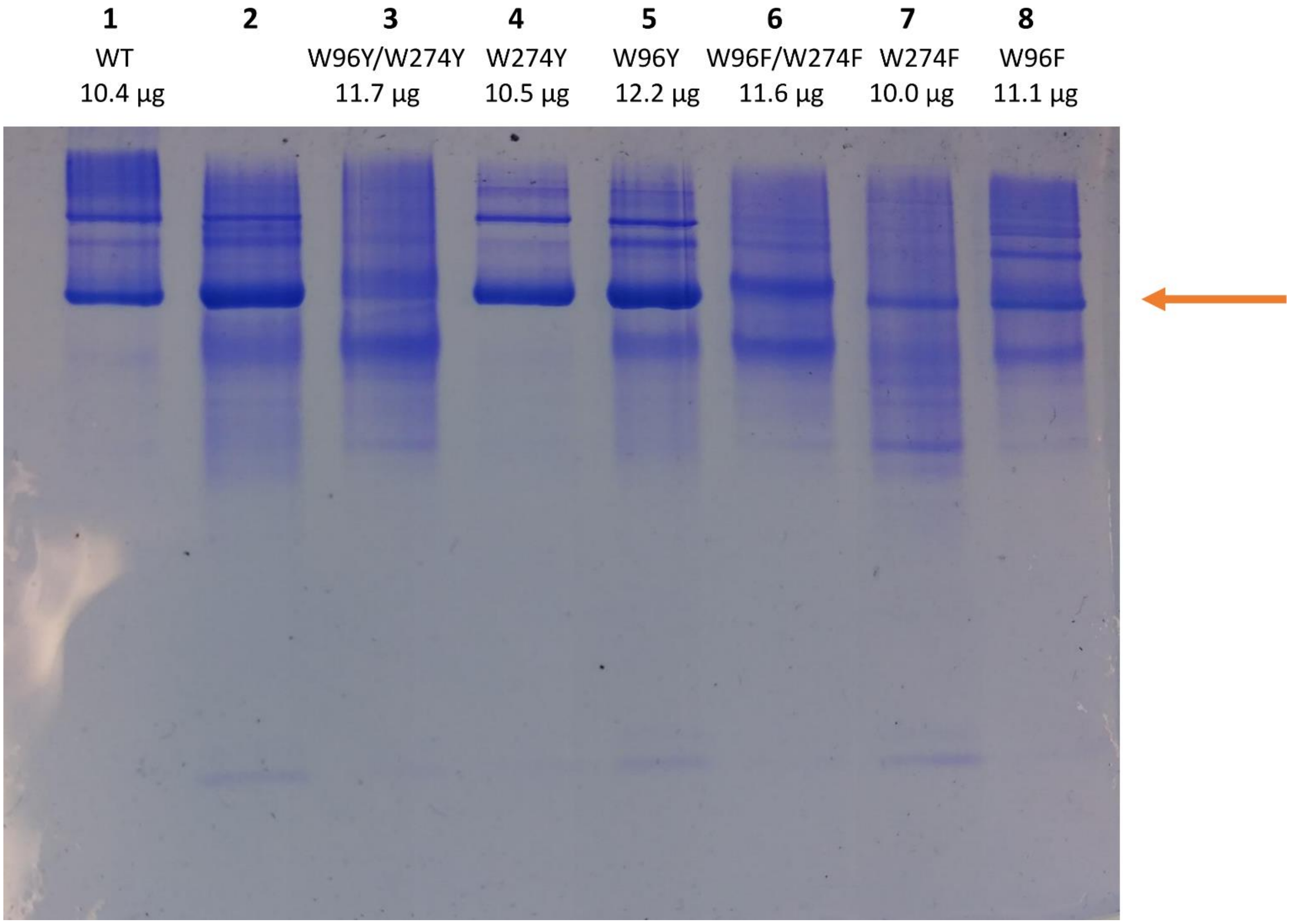

Figure S3: *Native PAGE of the mutants used in this study (lanes 3 – 8) compared with WT (lane 1). Lane 2 is for an OxDC mutant not discussed in this paper which is therefore not labeled. The resolving gel is formed from 8% acrylamide (v/w) and was run at 4 °C with 150 V. All mutants possess a band similar to the main band of WT as indicated by the orange arrow. Soluble aggregates of WT and other mutants have been previously reported in the literature via size exclusion chromatography (2). The phenylalanine mutants and the double mutants showed more smearing on the gel but also possess lower Mn content. Both double mutants show two bands despite W96Y/W274Y only having measurable activity.*

The phenylalanine mutants showed weaker hexameric bands and more smearing compared to the WT. However, these mutants possess lower Mn content compared to WT and the tyrosine mutants also. Moreover, even WT enzyme show significant smearing toward higher masses and it is well known that oxalate decarboxylase forms soluble aggregates in solution that are larger than hexamers (3,4).

Table SI-1. List of Primers for Mutagenesis.

Mutation	Forward 5' to 3'	Reverse 5' to 3'
W96F	GCGATTCGCGAGCTTCACTTCACTTCCATAAA	GTGAAGCTCGCGAATCGC
W96Y	CGAGCTTCACTACCATAAAGAAGCTGAATGGG	CGAATCGCGCCTGGCTTC
W274F	AGAACTGCACTTCCACCCGAATACCC	CTCATGGCGCCGGGTTCT
W274Y	AGAACTGCACTACCACCCGAATACCCAC	CTCATGGCGCCGGGTTCT

References:

1. Williams, C. J., Headd, J. J., Moriarty, N. W., Prisant, M. G., Videau, L. L., Deis, L. N., Verma, V., Keedy, D. A., Hintze, B. J., Chen, V. B., Jain, S., Lewis, S. M., Arendall, W. B., Snoeyink, J., Adams, P. D., Lovell, S. C., Richardson, J. S., and Richardson, D. C. (2018) MolProbity: More and better reference data for improved all-atom structure validation. *Protein Sci.* **27**, 293-315
2. Tanner, A., Bowater, L., Fairhurst, S. A., and Bornemann, S. (2001) Oxalate decarboxylase requires manganese and dioxygen for activity - Overexpression and characterization of Bacillus subtilis YvrK and YoaN. *Journal of Biological Chemistry* **276**, 43627-43634
3. Reinhardt, L. A., Svedružić, D., Chang, C. H., Cleland, W. W., and Richards, N. G. J. (2003) Heavy atom isotope effects on the reaction catalyzed by the oxalate decarboxylase from Bacillus Subtilis. *Journal of the American Chemical Society* **125**, 1244-1252
4. Just, V. J., Burrell, M. R., Bowater, L., McRobbie, I., Stevenson, C. E. M., Lawson, D. M., and Bornemann, S. (2007) The identity of the active site of oxalate decarboxylase and the importance of the stability of active-site lid conformations. *Biochem. J.* **407**, 397-406
